# SIRT1^+^ Adipose Derived Mesenchymal Stromal Stem Cells (ASCs) Suspended in Alginate Hydrogel for the Treatment of Subchondral Bone Cyst in Medial Femoral Condyle in the Horse. Clinical Report

**DOI:** 10.1007/s12015-020-10025-6

**Published:** 2020-08-15

**Authors:** Paweł Golonka, Katarzyna Kornicka-Garbowska, Krzysztof Marycz

**Affiliations:** 1 Equine Clinic Equivet, Gęsice 8, Domaniów, 55-216 Poland; 2 Department of Experimental Biology, Wroclaw University of Environmental and Life Sciences, Norwida 27B Street, A7 building, Wroclaw, 50-375 Poland; 3 International Institute of Translational Medicine, Malin, Jesionowa 11, Wisznia Mała, 55-114 Poland; 4 Faculty of Veterinary Medicine, Equine Clinic-Equine Surgery, Justus-Liebig-University, Giessen, 35392 Germany

**Keywords:** Horse, Injury, Adipose stem cells, Subchondral bone cyst

## Abstract

Stem cell based therapy are now commonly applied in human and veterinary medical practice especially in orthopaedics. Mesenchymal stromal stem cells isolated from adipose tissue (ASC) are first choice option due to relatively non-invasive and safe procedure of tissue harvesting. However, ASC therapeutic potential strongly rely on patients general health condition, age and life-style. For that reason, to enhance therapeutic potential of cells, they are modified *in vitro* using different approaches. Previous studies have shown, that ASC treated with resveratrol, herein called SIRT+, are characterised by decreased senescence, increased proliferation rate and improved clinical outcome in autologous therapies. Herein, SIRT + cells in alginate hydrogel were applied to 5 years old warm breed mare was clinically evaluated due to the left hind lameness due to subchondral bone cyst. The therapeutic effect was assessed by the analysis of lameness score and radiological evaluation. This case report demonstrates the therapeutic potential of SIRT + cells in the treatment of orthopaedics disorders in horses as complete bone remodelling occurred after therapy and horse came back to training.

## Introduction

Subchondral cystic lesions (SCLs) are referred in the literature and common veterinary practice as the subchondral bone cysts (SBCs). SBCs usually accompany the developmental joint disease complex and osteochondrosis (OC), however their inflammatory origin is underlined by some of the Authors as well [1–3]. While the OC lesions occur at the transition from the weight- bearing to the non-weight-bearing articular surface, SBCs are mostly found in weight-bearing area of the joint [4]. Located in femur condyle they are often large in size. Both, fibrous tissue harvested from the center of the lesion and the cystic fluid contain augmented levels of pro-inflammatory mediator prostaglandin E2 (PGE2) [5]. Furthermore, the up-regulation of interleukin 1 (IL-1) occurs at the periphery of the lesion while IL-6 at its center [6]. Increased concentration of IL-1 and IL-6 are common features in clinical cases of pathologic bone resorption and inflammation [7]. SBCs occur mainly in medial femoral condyle (45,8% of 703 lesions) [4] causing lameness in affected horses as a result of increased intracystic or intraosseus pressure [8]. If SBCs is associated with osteoarthritis, the joint cartilage is usually damaged as well. Conservative treatment of SBCs lesions using non-steroidial anti-inflammatory drugs (NSAIDs) was shown to be successful only in 33% of cases [4]. Surgical debridement of the lesion with a curettage of the cystic tissue and lining is considered the treatment of choice as a postoperative soundness up to 74% can be expected [4, 9] .

Mesenchymal stem cells (MSCs) are more and more frequently applied in clinical practice especially in orthopedic patients [10]. Since FDA approved equine model for human cellular therapies, stem cells application in horses might deliver valuable information regarding safety and efficacy of cellular based therapies [11]. MSCs pro-regenerative properties are explained by their paracrine activity, immunomodulatory properties and multilineage differentiation potential [12–14]. However aging, metabolic syndrome or type 2 diabetes have been showed to negatively affect MSC cytophysiological properties and limit their therapeutic value [15]. It was showed, that aging and endocrine disorders cause extensive generation and accumulation of reactive oxygen species (ROS), impairment of mitochondrial dynamics e.g. fusion and fission as well as induce excessive DNA methylation which reduce pro-regenerative potential of MSCs [16, 17]. For that reason, development of strategies able to reverse the aged phenotype of MSC in order to enhance their therapeutic potential are strongly desirable. Our group have developed the pharmacotherapy of metabolically-impaired MSC based on the application of resveratrol (RES) and 5-azacitidine (5‐AZA) which increased proliferative activity, improved osteogenic differentiation potential while decreased accumulation of free radical and DNA methylation status restoring MSC their therapeutic potential [18, 19].

It was reported, that RES activates the Sirtuin-1 (SIRT1), which inhibits NF-κB signaling by deacetylating the p65 subunit of NF-κB complex and thus protects the cells against apoptosis [20]. SIRT1 deacetylates and inactivates hypoxia-inducible factor (HIF)-1α, thus suppresses the genes expression targeted by HIF-1α in certain conditions. Moreover, it was showed that SIRT1 positive stem cells protects against bone loss by FOXO3a deacetylation and oxidative stress inhibition, however in mice model [21]. In turn, 3D culture of MSCs in alginate hydrogel was shown to improve their viability, metabolic activity as well as differentiation potential, shedding a promising light for its application in orthopedic practice. In our previous research we showed, that alginate hydrogel combined with ASCs significantly improved healing process in subchondral bone cysts [22]. Here, we combined SIRT1^+^ MSCs with alginate hydrogel for subchondral bone cyst treatment in medial femoral condyle in the horse. We believe, that the combination of SIRT1 + stem cells and 3D biodegradable matrix might become an effective strategy for subchondral bone cyst treatment.

## Matherials and Methods

### Case Description

5 years old warm breed mare was clinically evaluated due to the left hind lameness (3/5). During clinical examination the effusion of stifle joint was palpated. The large SBC in medial femoral condyle of left stifle was diagnosed in the course of radiological investigation. The horse was prepared for arthroscopic surgery of the stifle. The mare was anesthetized in dorsal recumbency for central arthroscopic approach, the technique developed by dr Karl Josef Boening [personal communication]. The leg was flexed such that the stifle and hock were at 90 degrees angle. The arthroscope was inserted through the middle patellar ligament halfway between the tibial crest and distal aspect of patella. Initially the joint distension was maintained with gas (CO_2_). This approach allows to penetrate femoropatellar and femorotibial joints. The skin portal for the instruments was created medial to medial patellar ligament. The communication between the joints was created by fenestration of the membrane dividing stifle into femoropatellar and medial and lateral compartments of femorotibial joint. The arthroscopic scissors were used for this purpose. Inspection of all compartments were done and the orifice to subchondral bone cyst was found in medial femoral condyle. Then, the third portal was created just above the SBC. Curette was inserted into the cyst and debridement was performed. Lavage with saline of all joints was done carefully after debridement and then CO2 was used for evacuation of fluid from the joint. Than the cyst was filled up with 3D complex of alginate hydrogel and SIRT1^+^ autologous adipose derived mesenchymal stem cells (ASCs) in a total volume of 2 ml. Skin incisions were than closed in a routine manner using Polyglactin 910 2 − 0. No special treatment was administered after the arthroscopy. The mare had confined to stall rest for three weeks, than started to walk in hand for next three weeks and walk under the saddle for another three weeks. Control radiographs were taken six months later.

### Adipose Tissue Harvesting

In order to isolate MSC from subcutaneous adipose tissue and isolate adipose-derived stem cells (ASCs), the area around the base of the tail was washed and shaved. Local analgesia was performed with 10 ml of a 2% lidocaine hydrochloride (lignocainum hydrochloricum WZF 2%, Polfa Warszawa S.A, Poland). Small piece of adipose tissue (2 g) was removed from the subcutis using a tweezers and scissors and the skin incision was sutured with non- absorbable suture material polyamide (Dafilon USP 1). Adipose tissue fragment was transferred to the laboratory in sterile Hank’s balanced salt solution (HBSS) supplemented with 1% penicillin/streptomycin (PS) on ice.

### Cell Isolation and Culture

Cells were isolated using mechanical-enzymatic method as described by our group previously [18]. Isolated cells were cultured in DMEM with 1 g/L glucose (DMEM LG) supplemented with 10% fetal bovine serum (FBS) and 1% PS. After the third passage, the culture medium was exchanged. In EMS I cells were cultured in medium containing 0.5 µM of AZA and 0.05 µM of RES while EMS II in 0.5 µM of AZA and 5 µM of RES for 24 h Cells were treated with AZA/RES for 24 h and then was detached from the dish, counted and prepared for injection. The molecular and functional characteristics of AZA/RES treated cells were previously published by our group [18, 19, 23]. DAPI, phalloidin stainings, scanning electron microscopy, proliferative analysis, efficiency of colony forming (CFU) and Ki67 analysis were performed as described elsewhere [18].

### Preparation of Sodium Alginate Hydrogel Combined with Stem Cells

Sodium alginate (2% w/v) was dissolved in 0.9% NaCl and sterilized. Then, 10 mln of ASCs were suspended in obtained solution and transferred to a syringe. Furthermore, the second syringe with sterile 10 mM CaCl2 solution was prepared in order to initialize polymerization of alginate in SBCs lesion.

### Evaluation of Lameness

The horse was presented for clinical evaluation due to left hindleg lameness. Lameness was first time observed 3 months earlier. The application of Rest and NSAiD therapy didn’t reduced lameness. The horse was grade 3/5 lame LH trotting straight both on hard and soft ground. Trotting on the circle the horse was more lame trotting to the right than trotting to the left direction. The flexion test was positive. The mild effusion of left stifle was palpated, right stifle was not effused. Local nerve block of tibial and fibular nerve were done in a routine manner. Result was negative, then the joint anesthesia was done: 20 ml of Lignocainum hydrochloricum 2% (Polfa Warszawa) was injected into the medial femoro tibial joint after aseptic preparation of the skin. Gait improvement was observed within 10 min after injection. The improvement was about 75%.

### Radiological Evaluation of Subchondral Bone Cyst

The radiographs of both hindlegs were done prior to local anaesthesia. Subchondral bone cyst was diagnosed in medial condyle of left femur in PA projection, so the local anaesthesia has served for confirmation of the source of pain.

### Anesthesia and Surgical Preparation

The horse was weighted prior to surgery with a weight of 500 kg body weight. For sedation Xylazine 1 mg/kg IV (Biowet Puławy) and Sedazin 20 mg/ml (Biowet Puławy) were applied in a total volume of 25 ml. Anesthesia was induced by diazepam (0.1 mg/kg IV) –(Relanium (Diazepamum, Polfa Warszawa 5 mg/ml 2 ml ampule (5 ampules were applied) combined with ketamine (2.2 mg/kg IV) (Bioketan, Vetoquinol) (total volume 11 ml). Anesthesia was maintained with the application of Isoflurane- 1000 mg/g (Isotek, Laboratorios Karizoo) (5%, later 3%) delivered in 5 L/ min oxygen (total infusion equaled 100 ml for 120 min anesthesia). Prior recovery, two ampules of 5 mg/ml Diazepam – Relanium (Diazepamum, Polfa Warszawa) were applied.

### Postoperative Management

The mare received Flunixin meglumine – (Vetaflunix VetAgro) (50 mg/ml) 15 ml iv for 3 consequtive days. Rest in the box for 2 weeks, and after this period walking in hand was implemented starting from 5 min twice daily. The horse was 3 weeks in the Hospital, than in the stable. Control of gait and radiographs were taken for long term once a months. The horse was back in normal work under the saddle 6 months after surgery.

## Results

### Characterization of SIRT + ASCs

**Fig. 1:**
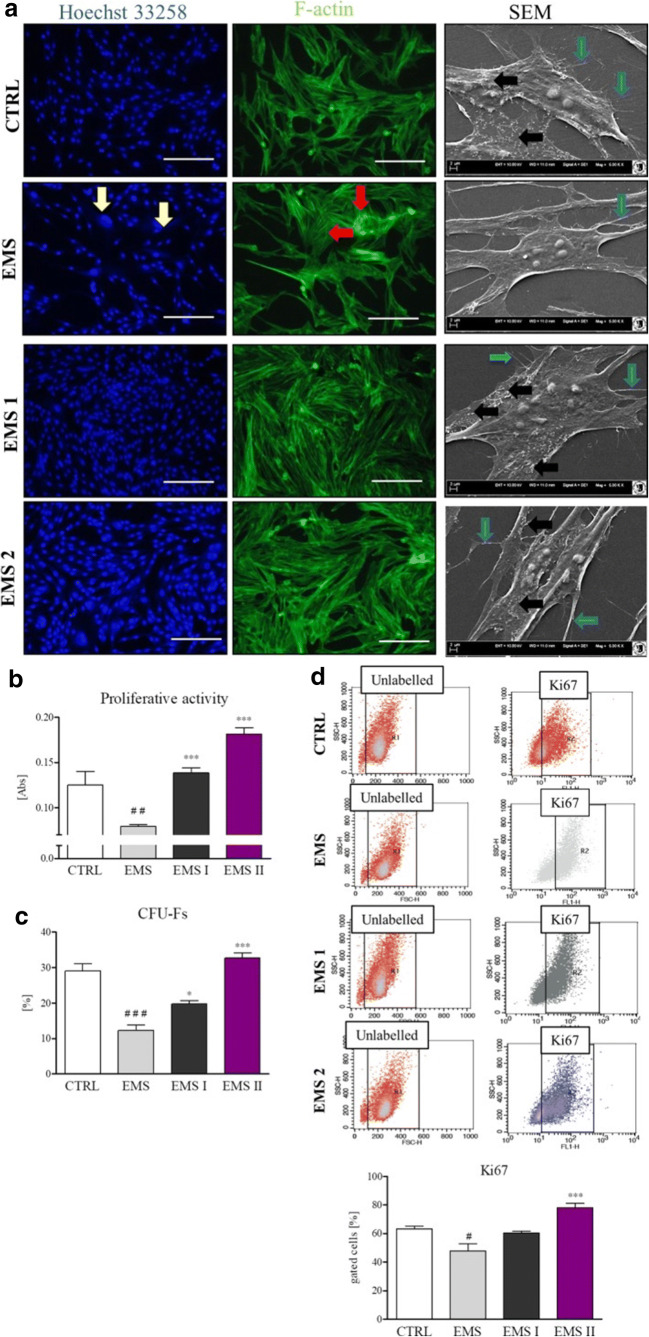
Morphology and growth pattern of SIRT + cells. SIRT + ASC (EMS I, EMS II) morphology was visualized by fluorescent and SEM imaging (**a**). Proliferation was established with using resazurin- based assay (**b**) and CFU‐E assay revealed the percentage of colonies consisting of more than 50 cells (**c**). Ki67 accumulation in cells was established with flow-cytometry. Results expressed as mean ± SD. #, *P < 0.05; ##P < 0.01; ###, *** P < 0.001. Statistical significance is indicated as asterisk (*) when comparing the result to ASC from EMS horse, and as hashtag (#) when comparing to ASC from healthy horse (CTRL). 1. (Reproduced from Kornicka et al. [18] under the Creative Commons Attribution License (https://onlinelibrary.wiley.com/doi/epdf/10.1111/jcmm.13914)

**Fig. 2:**
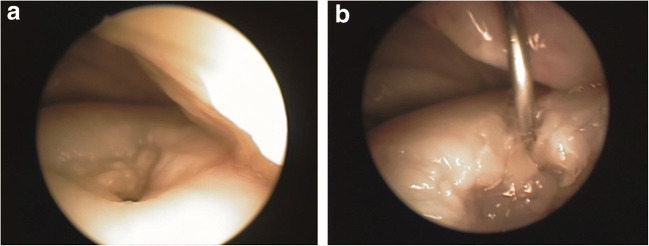
Arthroscopic view of the orifice of SBC in medial femoral condyle (**a**) and view of the cyst after debriment and fulfilled with alginate hydrogel and SIRT1^+^ ASCs stabilised with CaCl_2_

EMS I cells were cultured in medium containing 0.5 µM of AZA and 0.05 µM of RES while EMS II in 0.5 µM of AZA and 5 µM of RES for 24 h. Cells treated with combination of AZA/RES- EMS I and EMS II, were characterized by increased proliferation potential in comparison to untreated cells (EMS). In our previous research we have shown, that cells treated with AZA/RES gained spindle-shaped morphology and the number of enlarged, apoptotic cells was decreased (Fig. 1a). Cells treated with 5 µM of RES were characterized by the greatest proliferative activity (Fig. 1b), CFU-Fs (Fig. 2c) and expression of Ki67 (Fig. 1d) and for that reason, that group was selected for the therapeutic application in horse as described in the section “case report”.

### Evaluation of Therapy Effectiveness

**Fig. 3:**
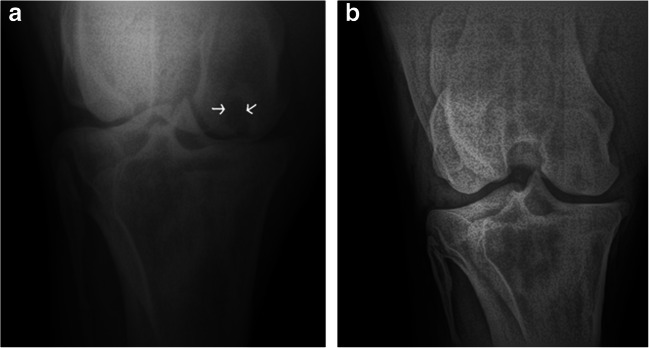
Radiographic evaluation. Radiograph taken prior to surgery. Note the large cystic lesion in medial femoral condyle (arrows) (**a**). Radograph six months after the surgery taken at the stable. There is still less than normal density of the bone visible in the condyle but articular surface remains intact (**b**)

Arthroscopy before the ASC application revealed of the orifice of SBC in medial femoral condyle (Fig. 2a). Cyst after debriment and fulfilled with alginate hydrogel combined with SIRT1^+^ ASCs stabilised with CaCl_2_ was also visualized (Fig. 2b). Postoperative radiographs showed remodeling of the cyst replaced by the bone tissue (Fig. 3a, b). The horse wasn’t lame and was back in normal work under the saddle within six months. Long term observation didn’t revealed any lameness and the mare is still used under the saddle.

## Discussion

Stem cell based therapies bring hope for the treatment of many diseases including musculoskeletal system disorders [24–26]. In this report we used 3 dimensional (3D) alginate hydrogel combined with SIRT1^+^ ASCs for subchondral bone cyst treatment in medial femoral condyle in the horse. We have found, that combination of both 3D alginate with SIRT1^+^ ASCs positively affects subchondral bone remodeling. The radiological evaluation performed after 6 months post transplantation showed bone remodeling and full patient recovery. Moreover, we observed no lameness what shed a promising light for that therapeutic strategy. Observed clinical effect in the current study might be explain by enhanced multipotency of ASCs since SIRT1 + cells exhibit anti-aging and anti-oxidative properties, which correspond with improved osteogenic differentiation potential [18, 19]. Recently, mesenchymal stem cells isolated from adipose tissue (ASCs) have been shown to possess unique therapeutic potential due to their multipotent characteristics, ability to differentiate into multilineages as well as their anabolic activity [12, 27–30]. In our previous clinical study, we showed, that combination of autologous ASCs with alginate hydrogel improves bone remodeling in subchondral bone cysts [22] without arthroscopic operation.

It was demonstrated, that incubation of ASCs with both AZA and RES improves multilineage differentiation potential including osteogenesis through improved mitochondrial metabolism and dynamics. The study performed by Kornicka et al. [18, 31] have shown, that RES possesses immunomodulatory and anti-inflammatory effect through activation of AMPK/PGC‐1α signaling – all critical for bone remodeling. Recently, Choi and colleges [32] has demonstrated in rat calvaria defect model, pro-regenerative effect of MSCs pretreated with RES. The beneficial effect has been explained by activation of SIRT1-SOX2 axis. In turn, pre treatment of MSCs has been showed to reverse aged phenotype, increased proliferative activity and improved osteogenic differentiation potential, while decreased the accumulation of oxidative stress factors and DNA methylation status [23]. Moreover, the study of Yan and colleges, showed, that MSCs pretreatment with AZA induced proliferation and improved osteogenic differentiation potential through increase of AP activity and matrix mineralization as well as with an increased TET2 and TET3 gene expression [33].

In the present study, for the first time we demonstrated, that pretreatment of ASCs with both AZA and RES lead to improvement of osteogenic differentiation potential *in vivo* when combined with 3D alginate hydrogel. Finally, application of SIRT1 + ASCs combined with 3D hydrogel for subchondral bone cysts treatment replace it with normal bone tissue and allows to full recovery from the disease.
